# Clinical Manifestations and Diagnostic Approach to Arrhythmogenic Right Ventricular Cardiomyopathy – A Case Report and Literature Review

**DOI:** 10.7759/cureus.11429

**Published:** 2020-11-10

**Authors:** Raja S Mushtaque, Rabia Mushtaque, Shahbano Baloch, Muhammad Idrees, Haseeb Bhatti

**Affiliations:** 1 Cardiology, National Institute of Cardiovascular Diseases, Karachi, PAK; 2 Internal Medicine, Jinnah Postgraduate Medical Center, Karachi, PAK; 3 Internal Medicine, Bassett Medical Center, Cooperstown, USA

**Keywords:** ventricular tachycardia (vt) storm, arrhythmogenic right ventricular dysplasia, implantable cardioverter-defibrillator, arvc, arvd, revised task force criteria 2010

## Abstract

Arrhythmogenic right ventricular cardiomyopathy (ARVC) is a rare inherited disorder, which is characterized by fibrofatty degeneration of cardiac muscles mainly in the right ventricular myocardium. It may cause tachyarrhythmias or right-heart failure or may cause sudden death, especially in young athletes. In our case report, we present a case of young age male patient who presented at a local community hospital with the complaint of atypical chest pain, palpitations, and vomiting and sustained ventricular tachycardia (VT) on electrocardiograph (ECG) showing sustained VT, left bundle branch morphology with the superior axis. The normal sinus rhythm was achieved after multiple direct current (DC) cardioversion attempts, and he was referred to our tertiary care hospital. Later ECG demonstrated epsilon waves and T wave inversion in V1 to V4 and right bundle branch block (RBBB) morphology. The echocardiography showed a severely dilated right ventricle with dysfunction and right ventricle ventricular apical aneurysm. The definitive diagnosis of ARVC was made as per Revised Task Force Criteria 2010, and the electrophysiology review suggested implantable cardiac defibrillator (ICD) device placement. The patient successfully received a dual-chamber ICD device, and he remained asymptomatic.

## Introduction

Background

Arrhythmogenic right ventricular cardiomyopathy (ARVC) also known as arrhythmogenic cardiomyopathy (ACM) or arrhythmogenic right ventricular dysplasia (ARVD) is a rare inherited autosomal dominant disorder with variable expression [1]. The prevalence of ARVC is recorded to be two to five per 10,000 persons [2,3]. Most cases of ARVC are being diagnosed before the age of 40 years [4], and it can result in sudden death, which is the most severe manifestation of ARVC, especially in young athletes. It is characterized by fibrofatty degeneration of cardiac muscles mainly in the right ventricular myocardium [5] impeding electrical conduction, causing tachyarrhythmias including ventricular tachycardia (VT) or supraventricular arrhythmias [6]. It may also present right-heart failure or maybe asymptomatic cardiomegaly [5]. The point mutations in genes encoding for desmosomal proteins have been found as the culprit in triggering the disease pathogenesis. More specifically, the mutation in the desmin (DES) gene could cause ARVC [7]. It has been found that exercise can worsen ARVC by increasing afterload and wall stress. It is also proposed that inflammatory or infectious conditions have also been precipitating the condition [8].

ARVC can be categorized into four stages that include the subclinical stage with covert functional and structural abnormalities, though sudden cardiac death (SCD) can occur in this stage. In the second stage, there are overt electrocardiogram findings of right ventricle (RV) arrhythmias along with both structural and functional abnormalities. In the third stage, there is severe right ventricular dysfunction and the absence of left ventricle (LV) involvement. In the fourth stage, there is biventricular involvement with severe right and left ventricle dysfunction [9]. ARVC is an uncommon disease, and thus identifying and diagnosing this disorder can be challenging and can also delay the treatment. Our case report summarizes the classical, clinical as well as diagnostic findings of ARVC and discusses management options.

## Case presentation

A 30-year-old male patient with no known comorbidities was shifted to tertiary care hospital after 12 hours following his initial presentation at a local community hospital with the complaint of atypical chest pain, palpitations, and vomiting for one day and sustained VT on ECG. During that presentation, the patient had recurrent episodes of VT storm) and the patient was also hypotensive, and normal sinus rhythm was achieved after multiple direct current (DC) cardioversion attempts (20-25 shocks of 200-270-300 J strength were given) and he was also given injection amiodarone 150 mg I/V Stat (documented in referral summary). After admission to our tertiary care hospital, the patient had no ventricular arrhythmic episodes. He denied any sudden death in his family. He also declined any drug intake history or any substance abuse. On examination, the young male patient, lying on the bed, well oriented with time, place, and person. He was vitally stable at the time of examination, and there was normal vesicular breath sounds bilaterally on chest auscultation while the first and the second heart sounds were audible of equal intensity without added murmurs on precordial auscultation.

His first ECG during the initial presentation reported sustained VT, left bundle branch morphology with the superior axis (mentioned in his referral documents). After stabilization, ECG showed epsilon waves and T wave inversion in V1 to V4 and right bundle branch block (RBBB) morphology. On further workup, the echocardiography showed a severely dilated RV with dysfunction, RV ventricular apical aneurysm, severe tricuspid regurgitation, and these findings were suggestive of ARVC. There was also moderate to severe generalized systolic dysfunction with a left ventricular ejection fraction of 30%. The specific findings of echocardiography are mentioned in Table 2. The left heart catheterization (LHC) was also done, which showed normal coronary arteries. The definitive diagnosis of ARVC was made as per Revised Task Force Criteria 2010. The electrophysiology review suggested for dual-chamber implantable cardiac defibrillator (ICD) device. He had successful ICD implantation and later on the patient was asymptomatic. The patient was discharged on home medicines including beta-blockers (bisoprolol 5 mg x OD), angiotensin II receptor blockers ([ARB] losartan potassium 50 mg HS], spironolactone 20 mg tablet x OD, and amiodarone 400 mg x HS.

**Figure 1:**
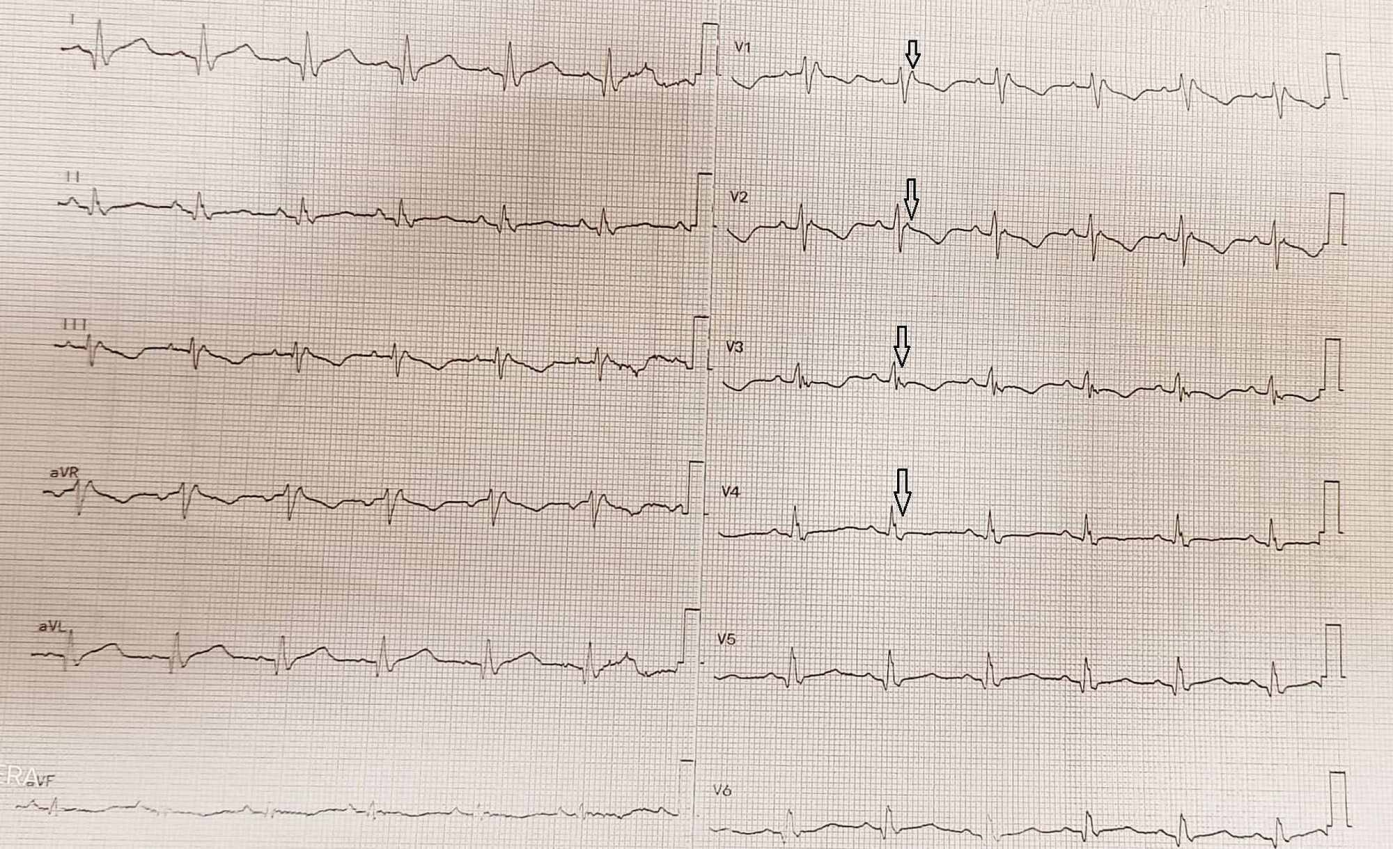
Electrocardiogram (ECG) showing epsilon waves and T wave inversion in V1 to V4 and RBBB morphology RBBB: Right bundle branch block.

**Figure 2:**
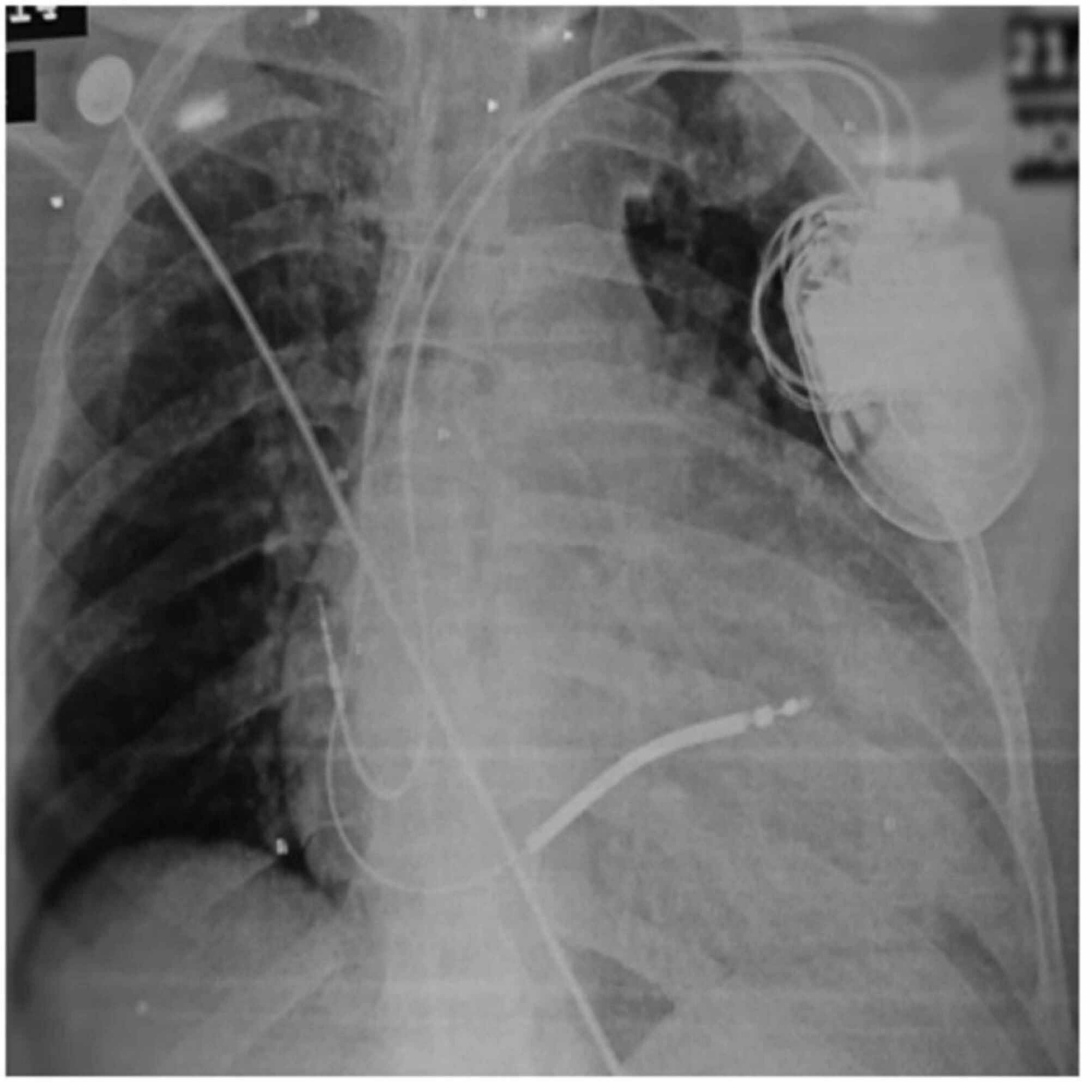
Post-ICD chest x-ray (AP-view) ICD: implantable cardiac defibrillator; AP: anteroposterior.

The basic laboratory workup of the patient is given in Table 1, whereas echocardiographic findings are mentioned in Table 2. Electrocardiogram (ECG) findings are illustrated in Figure 1, and post ICD x-ray is shown in Figure 2.

**Table 1:** Basic laboratory workup Hb: Hemoglobin; MCV: mean corpuscular volume; TLC: total leukocyte count; Cr: creatinine; Na: sodium; K: potassium; PT: promthrombin time; INR: international normalization ratio; HbA1c: hemoglobin A1c.

Laboratory Investigations	Results	Normal Values
Hb	16.3	14.0-17.4 g/dl
MCV	83.2	76.5-96 fl
TLC	13.0	5.00-10.00 x 10 x 9/L
Neutrophils	62%	50-75%
Lymphocytes	33%	25-40%
Platelets	300	140-400 x 10 x 9/L
Urea	41	10-50 mg/dl
Cr	1.1	0.5-1.2
Na	138	136-149 mEq/L
K	4.2	3.50-5.50 mEq/L
Troponin I	2.67	0.0572 ng/ml
PT	12.8	9.3-14.0 seconds
INR	1.2	0.8-1.2
HbA1c	5.6%	Below 6.0%

**Table 2:** Specific echocardiographic findings RV: Right ventricle; TAPSE: tricuspid annular plane systolic excursion; PLAX: parasternal long axis; PSAX: parasternal short axis; RVOT: right ventricle outflow tract; LVEF: left ventricle ejection fraction.

RV Size	60 mm
TAPSE	10 mm
PLAX RVOT	48 mm
PSAX RVOT	36 mm
LVEF	30%

## Discussion

Initially, international task force criteria for the clinical diagnosis of ARVC were published in 1994 based on structural, histological, ECG changes, and familial features of the disease and these features were subdivided into major and minor categories. That criterion was highly specific focusing on symptomatic cases and SCD victims but lack sensitivity for the early disease [10]. Thus, in 2010 a revised version was formulated to improve the diagnosis and management of this disorder [10], this criterion include six categories further divided into major or minor classes, mentioned in Table 3.

**Table 3:** Revised Task Force Criteria 2010 2D: two dimensional; PLAX: parasternal long axis; PSAX: parasternal short axis; RVOT: right ventricle outflow tract; MRI: magnetic resonance imaging; BSA: body surface area; RV: right ventricle; RVEF: right ventricle ejection fraction; RBBB: right bundle branch block; SAECG: signal-averaged electrocardiography; ARVC: arrhythmogenic right ventricular cardiomyopathy; aVF: augmented vector foot; aVL: augmented vector left. Diagnostic terminology for revised criteria Definite diagnosis: Two major or one major and two minor criteria or four minor from different categories Borderline: One major and one minor or three minor criteria from different categories Possible: One major or two minor criteria from different categories (a) Hypokinesis is not included in this or subsequent definitions of RV regional wall motion abnormalities for the proposed modified criteria. (b) A pathogenic mutation is a DNA alteration associated with ARVC that alters or is expected to alter the encoded protein, is unobserved or rare in a large non-ARVC control population, and either alters or is predicted to alter the structure or function of the protein or has demonstrated linkage to the disease phenotype in a conclusive pedigree.

1. Global or regional dysfunction and structural alterations on echocardiography, CMRI, and/or RV angiography (a)	
Major	2D echocardiography: Regional RV akinesia or dyskinesia or aneurysm and one of the following (end-diastole): PLAX RVOT = 32 mm (= 19 mm/m^2^ body surface area [BSA] corrected); PSAX RVOT > 36 mm (= 21 mm/m^2^ BSA corrected); fractional area change = 33%.
	Cardiac MRI: Regional RV akinesia or dyskinesia or dyssynchronous RV contraction and one of the following: Ratio of RV end-diastolic volume to BSA in males = 110 mL/m^2^ or BSA = 100 mL/m^2^ in females; RVEF = 40%.
Minor	2D echocardiography: Regional RV akinesia or dyskinesia and one of the following: PLAX RVOT = 29 to < 32 mm (= 16 to < 19 mm/m^2^ BSA corrected); PSAX RVOT = 32 to < 36 mm (= 18 to <21 mm/m^2^ BSA corrected); fractional area change >33% to =40%.
	Cardiac MRI: Regional RV akinesia or dyskinesia or dyschronous RV contraction and one of the following: Ratio of RV end-diastolic volume to BSA = 100 to <110 mL/m^2^ in males or BSA = 90 to <100 mL/m^2^ in females; RVEF >40% to =45%.
2. Tissue characterization of wall	
Major	Residual myocytes < 60% by morphometric analysis (or < 50% by estimation), with the fibrous replacement of the RV free wall myocardium in =1 sample, with or without fatty replacement of tissue.
Minor	Residual myocytes 60%-75% by morphometric analysis (or 50%-65% by estimation), with the fibrous replacement of the RV free wall myocardium in =1 sample, with or without fatty replacement of tissue.
3. Repolarization abnormalities	
Major	Inverted T waves in right precordial leads (V1-V3) or beyond in individuals older than 14 years in the absence of complete RBBB.
Minor	Inverted T waves in leads V1 and V2 in individuals older than 14 years in the absence of complete RBBB or V4, V5, or V6 inverted T waves in leads V1, V2, V3, and V4 in individuals older than 14 years in the presence of complete RBBB.
4. Depolarization/conduction abnormalities	
Major	Epsilon waves (low-amplitude signals between the end of QRS complex to the onset of T wave) in leads V1-V3.
Minor	SAECG should have at least one of three parameters in the absence of QRS duration of 110 ms or more on standard ECG. These parameters include the following: Filtered QRS duration = 114 ms; duration of terminal QRS < 40 µV (low-amplitude signal duration) = 38 ms; root-mean-square voltage of terminal 40 ms =20 µV.
5. Arrhythmia	
Major	Nonsustained or sustained ventricular tachycardia of left bundle branch morphology with the superior axis (negative or indeterminate QRS in leads II, III, and aVF and positive in lead aVL.
Minor	Nonsustained or sustained ventricular tachycardia of RV outflow configuration left bundle branch block morphology with the inferior axis (positive QRS in leads II, III, and aVF and negative in lead aVL).
6. Family history	
Major	ARVC confirmed in a first-degree relative who meets current Task Force Criteria. ARVC confirmed pathologically at autopsy or surgery in a first-degree relative. Identification of a pathogenic mutation (b) categorized as associated or probably associated with ARVC in the patient under evaluation.
Minor	History of ARVC in a first-degree relative in whom it is not possible or practical to determine whether the family member meets current Task Force Criteria Premature sudden death (<35 years of age) due to suspected ARVC in a first-degree relative. ARVC confirmed pathologically or by current Task Force Criteria in a second-degree relative.

In our case report, the patient met three major criteria of the revised task force for ARVC 2010, thus making a definitive diagnosis of ARVC. The patient had a severely dilated right ventricle (60 mm) with dysfunction, right ventricle ventricular apical aneurysm on 2D echocardiography, while PLAX RVOT was 48 mm and PSAX RVOT was 36 mm. On ECG, major depolarization/conduction abnormalities criteria were met, as epsilon waves in V1 to V4 leads were noticed. The patient also fulfilled arrhythmic major criteria as he had sustained VT with left bundle branch morphology with a superior axis during his initial presentation. We also noticed that patient was categorized into the fourth stage of ARVC as he had biventricular involvement.

The management options for patients with ARVC include lifestyle changes, pharmacological treatment, catheter ablation, ICD placement, or heart transplantation [11]. In this patient, ICD device was a class I recommendation; implantation of an ICD is recommended in ARVC patients who have experienced ≥1 episode of hemodynamically unstable sustained VT or VF (class I), or implantation of an ICD is recommended in ARVC patients with severe systolic dysfunction of the RV, LV, or both, irrespective of arrhythmias (class I) [11]. The patient received a dual-chamber ICD after he was 48 hours free of ventricular arrhythmia for secondary prevention. He was observed in the hospital for two days, and no post-procedural complications were noticed. He was advised to avoid any strenuous activities that can cause palpitations or syncope. Competitive sports activity has been shown to increase the risk of SCD by five-fold in adolescents and young adults with ARVC [12].

The pharmacological treatment for ARVC includes β-blockers, heart failure therapy, or antiarrhythmic agents. Beta-blocker therapy is recommended in patients with recurrent VT, appropriate ICD therapies, or inappropriate ICD interventions resulting from sinus tachycardia, supraventricular tachycardia, or atrial fibrillation/flutter with the high-ventricular rate (class I). Beta-blocker therapy should also be considered in all patients with ARVC irrespective of arrhythmias (class IIa). Patients who have developed right- or left-sided heart failure or biventricular failure should receive standard pharmacological treatment with angiotensin-converting-enzyme inhibitors, angiotensin II receptor blockers, β-blockers, and diuretics as per clinical assessment (class I). Antiarrhythmic agents are recommended as an adjunct therapy to ICD in patients with frequent appropriate device discharges (class I). [11].

## Conclusions

ARVC is a rare disorder, and correctly diagnosing is important to avoid unnecessary delay in managing the patient. Revised task force criteria published in 2010 outlines all aspects to diagnose ARVC. SCD is the most feared complication of ARVC; thus, prompt diagnosis and offering ICD placement could save lives. This case report enlightens the clinical and the diagnostic investigation findings in our patient as well as illustrates ARVC literature to understand the pathophysiology of the disease and diagnostic criteria.
